# Synergistic Effects of Marine Fish and Insect-Derived Proteins on Honey Bee (*Apis mellifera* L.) Health, Longevity, and Gut Microbiota

**DOI:** 10.3390/insects17070741

**Published:** 2026-07-20

**Authors:** Khanchai Danmek, Chuleui Jung, Tippapha Pisithkul, Pornprapa Saenluang, Sukjun Sun, Hyeonjeong Jang, Sampat Ghosh, Phonkrit Maniwara, Pichet Praphawilai, Bajaree Chuttong

**Affiliations:** 1 Biotechnology Program, School of Agriculture and Natural Resources, University of Phayao, Phayao 56000, Thailand; khanchai.da@up.ac.th (K.D.); pornprapa.sa@up.ac.th (P.S.); 2 Department of Plant Medicals, Gyeongkuk National University, Andong 36729, Republic of Korea; scv6309@naver.com (S.S.); jhj971008@naver.com (H.J.); 3 Agricultural Research Institute, Gyeongkuk National University, Andong 36729, Republic of Korea; 4 Program in Biotechnology, Faculty of Science, Maejo University, Chiang Mai 50290, Thailand; tpisithkul@gmail.com; 5 Department of Life Science, Sardar Patel University, Balaghat 481331, Madhya Pradesh, India; sampatghosh.bee@gmail.com; 6 Postharvest Technology Research Center, Faculty of Agriculture, Chiang Mai University, Chiang Mai 50200, Thailand; maniwara016@gmail.com; 7 Office of Research Administration, Chiang Mai University, Chiang Mai 50200, Thailand; pichet.p@cmu.ac.th; 8 Meliponini and Apini Research Laboratory, Department of Entomology and Plant Pathology, Faculty of Agriculture, Chiang Mai University, Chiang Mai 50200, Thailand

**Keywords:** *Apis mellifera*, artificial pollen diet, pollination services, nutritional stress mitigation, gut microbiome, marine protein

## Abstract

Pollen limitation compromises honey bee health and pollination services, and existing pollen substitutes are typically formulated around crude protein content while lacking the long-chain omega-3 fatty acids naturally present in pollen and bee-collected re-sources. To address both protein and fatty acid gaps in current substitutes, this study evaluated marine-protein and insect-larvae-based diets. We assessed how formulations using Asian seabass (*Lates calcarifer*) and insect larvae affect the nutrition, physiology, longevity, and gut microbiota of *Apis mellifera* L. Newly emerged bees were fed for 35 days on: sugar syrup (negative control), sugar syrup and natural pollen (positive control), or four experimental diets: seabass-based (SB), or SB supplemented with meal-worm (SBM), or wax moth (SBW), or black soldier fly (SBB). Experimental diets (12.48–15.59% crude protein, vs. 17.24% in natural pollen) while SB supplied eicosapentaenoic acid (39.3–53.3 mg/100 g) and docosahexaenoic acid (37.2–52.3 mg/100 g), and SBB was additionally rich in lauric acid (604.3 mg/100 g). SBB- and SBM-fed bees exhibited the greatest hypopharyngeal gland development (0.123 mm and 0.115 mm, respectively, vs. 0.060 mm in sugar-only controls), with no significant difference from SB alone. All SB-based diets supported significantly greater survival than the sugar-only control (log-rank *p* < 0.0001) and did not differ significantly from the natural pollen treatment with SBB showing the highest and most consistent day-35 survival (81.1%). All sea-bass-based diets significantly altered the gut microbial community structure, promoting short-chain fatty acid (SCFA)-associated bacteria such as *Faecalibacterium prausnitzii* and *Blautia wexlerae.* This enrichment may reflect not only the omega-3 and lauric acid content of the diets but also the presence of substrates that can be utilized by gut microbes, which may exert prebiotic-like effects by favoring SCFA-producing bacteria. These results demonstrate that marine and insect-derived nutrients can supply protein while also providing beneficial lipids and microbially utilizable substrates that promote the enrichment of beneficial SCFA-associated bacteria not typically dominant in the honey bee gut. These enriched diets, particularly SBB, are promising candidates war-ranting further evaluation at the colony and field levels.

## Simple Summary

Pollen scarcity represents a significant threat to honey bee health and undermines vital agricultural pollination services. To mitigate this issue, this study aimed to develop alternative artificial diets incorporating marine fish (Asian seabass) and insect larvae (such as black soldier fly). These formulations were evaluated to determine their effects on the survival, physiological development, and gut health of young worker bees. The results demonstrated that combining marine and insect-derived proteins created a synergistic nutritional profile. This combination significantly enhanced bee longevity and accelerated the development of the key glands responsible for processing nutrients and producing brood food. Additionally, these specialized diets promoted the growth of beneficial, health-associated digestive bacteria within the honey bee gut. These findings indicate that marine and insect resources can serve as highly effective, functional ingredients in honey bee nutrition. These findings identify marine- and insect-derived protein hydrolysates as promising candidate ingredients for future colony-level and field trials aimed at alleviating nutritional stress.

## 1. Introduction

Insect pollination underpins global agricultural productivity, biodiversity, and food security [1,2]. Pollinator-dependent crops, particularly high-value fruits and vegetables, contribute significantly to the global economy [3,4]. The Western honey bee (*Apis mellifera* L.), the primary managed pollinator worldwide, supports approximately one-third of global food production through commercial crop pollination [5,6,7]. Consequently, maintaining honey bee health is essential for sustainable and resilient food systems [5,8]. However, pollen limitation—the scarcity of high-quality natural pollen—represents a fundamental nutritional constraint that threatens colony sustainability and the ecosystem services they provide [1,9]. As pollen is the sole source of essential proteins, lipids, vitamins, and minerals required for brood development and physiological function, its absence cannot be compensated for by sugar syrup supplementation alone [10,11,12]. Nutritional stress arising from pollen scarcity compromises immune function, reduces brood production, and increases vulnerability to pathogens, often leading to colony collapse [10]. Gut microbiota composition plays a central role in mediating these effects, contributing to nutrient metabolism, immune priming, and resistance to opportunistic pathogens in honey bees [13,14]. Diet composition and nutrient intake are known to shape gut microbial community structure and diversity in honey bees [15,16], including our own previous work showing that the diet fed to honey bees and the nutrients they ingest influence gut microbiota diversity [17]. Shifts toward short-chain-fatty-acid-producing bacterial taxa have been proposed as one mechanistic pathway linking dietary protein source to physiological outcomes such as glandular development and longevity [15], providing the rationale for evaluating gut microbial community shifts alongside physiological indicators in this study.

To mitigate these impacts, artificial pollen diets have been studied for decades [18]. Early research focused on simple protein supplementation, but modern formulations emphasize a “nutritionally balanced” approach to better mimic natural pollen, prioritizing nutrient quality and amino acid profiles over crude protein quantity [19,20]. These diets are categorized as either supplements (pollen mixed with other ingredients) or substitutes (pollen-free). While many current substitutes rely on plant-based proteins, yeast, or algae [15,21,22], they often lack the complex lipid profiles, specifically essential polyunsaturated fatty acids (PUFAs), which are found in natural pollen [23,24]. Previous studies emphasize that omega-3 and omega-6 fatty acids are vital for honey bee development, metabolic regulation, and immune resilience [24,25]. Therefore, identifying alternative ingredients that bridge this lipid gap is critical for next-generation pollen substitutes. In the context of broader global shifts, climate change and agricultural intensification cause seasonal floral gaps (pollen dearth periods), directly leading to severe nutritional stress for honey bees. Consequently, developing an effective marine-insect diet serves not merely as an alternative food source, but as a crucial climate-adaptation management tool to maintain colony resilience and secure pollination services when natural landscapes fail.

Marine fish species represent a promising but underutilized high-quality protein and lipid source, characterized by high digestibility and rich omega-3 fatty acid content [26,27]. While marine fish hydrolysates have shown beneficial effects when added to liquid feeds [28], their efficacy as a primary component in solid pollen substitutes remains largely unexplored. In parallel, insect-derived ingredients, specifically larvae of mealworms (*Tenebrio molitor*), wax moths (*Galleria mellonella*), and black soldier flies (*Hermetia illucens*), are rich in omega-6 fatty acids and proteins that closely mimic the nutritional density of natural pollen [29]. We suggest that the complementary fatty acid profiles of marine fish (omega-3 rich) and insect larvae (omega-6 rich) provide a synergistic nutritional balance that plant-based substitutes often lack. While some research suggests that animal-derived proteins may be less palatable or difficult for bees to process compared to plant proteins, we hypothesized that the unique lipid–protein complex of marine and insect biomass could offset these concerns. While the use of animal-derived proteins in honey bee diets is not new, prior studies have tested fish-based [22,23,24] or insect-based [25] ingredients in isolation; to our knowledge, no study has evaluated a combined marine fish–insect larvae hydrolysate as a single formulation.

This study evaluated the effects of novel artificial pollen diets formulated with Asian sea bass (*Lates calcarifer*) and insect-derived ingredients on honey bee health and longevity. We hypothesized that these animal-protein-enriched diets would (1) enhance physiological health indicators, such as hypopharyngeal gland development, (2) extend lifespan compared to sugar-only controls, and (3) promote beneficial shifts in the gut microbiome by providing novel fermentative substrates. Our findings indicate that the inclusion of black soldier fly larvae in a seabass-based diet significantly improves physiological development and recruits beneficial fermentative bacteria, demonstrating that animal-derived nutrient complexes can effectively replace natural pollen. By testing these nutritionally targeted formulations, this work seeks to identify candidate ingredients for colony maintenance during periods of natural pollen dearth, to be validated in future colony-level and field-scale studies.

## 2. Materials and Methods

### 2.1. Preparation of Ingredients and Samples

Asian sea bass (*Lates calcarifer*) was purchased from local supermarkets in Chiang Mai Province, Thailand, in October 2025. Fish declared as freshly harvested within 24 h were selected to limit quality degradation and reduce the likelihood of microbial contamination. Prior to processing, the fish were manually processed by removing scales, heads, skeletal structures, and visceral organs, with only the skin, adipose tissue, and muscle retained for further use.

Larvae of mealworm (*Tenebrio molitor*), wax moth (*Galleria mellonella*), and black soldier fly (*Hermetia illucens*) were obtained from the laboratory-rearing facility of the Department of Entomology and Plant Pathology, Faculty of Agriculture, Chiang Mai University, Chiang Mai, Thailand (19.07262° N, 99.88434° E) in October 2025. All insect samples were reared, harvested, and handled in accordance with standardized laboratory husbandry and biosafety protocols. All raw animal biomass samples were stored at 4 °C prior to enzymatic pretreatment to preserve nutrient freshness and prevent deterioration. In addition, a commercially available mixed pollen was purchased from a local apicultural supply provider in Chiang Mai Province, Thailand, to serve as a baseline formulation component.

### 2.2. Experimental Diet Formulation

Raw animal tissues (seabass and insect larvae) were sealed in heat-resistant plastic bags and treated with 2.0% (*w*/*w*) of a commercial acid protease (iKnowZyme™, Reach Biotechnology Co., Ltd., Bangkok, Thailand). Enzymatic hydrolysis was carried out in a water bath maintained at 60 °C for 3 h, after which the reaction was terminated by immersing the samples in boiling water for 15 min. The resulting hydrolyzed materials were homogenized to a uniform consistency to yield pretreated protein substrates.

Six experimental treatments were prepared: (1) Syrup Control (SR): A negative control consisting solely of a 50% (*w*/*w*) sucrose syrup solution without solid diet supplementation. (2) Mixed Pollen (MP): A positive control prepared by blending natural mixed pollen powder with 50% (*w*/*w*) sucrose syrup at a 2:1 ratio. (3) Seabass-Based Diet (SB): Formulated with pretreated fish hydrolysate at 51.9 g/100 g of diet as the exclusive animal protein source, without insect larvae. (4) Seabass–Mealworm Diet (SBM): Formulated by replacing a portion of the fish substrate with *T. molitor* larvae at a fixed inclusion level of 20 g/100 g of diet. (5) Seabass–Wax Moth Diet (SBW): Formulated by replacing a portion of the fish substrate with *G. mellonella* larvae at a fixed inclusion level of 20 g/100 g of diet. And (6) Seabass–Black Soldier Fly Diet (SBB): Formulated by replacing a portion of the fish substrate with *H. illucens* larvae at a fixed inclusion level of 20 g/100 g of diet.

Across all four seabass-containing diets (SB, SBM, SBW, and SBB), the concentrations of background ingredients were held constant: mixed pollen powder (10 g/100 g), sugar (35 g/100 g), vitamin–mineral premix (0.1 g/100 g), xanthan gum (1 g/100 g), and rum (40% alcohol; 2 g/100 g). The total weight of each formulation batch was adjusted to 100 g to ensure physical homogeneity. Diets were processed using microwave heating for 10 min and transferred into specialized molds to produce standardized solid diet patties for colony presentation.

### 2.3. Proximate Composition and Mineral Analysis

The proximate nutrient composition of the experimental diets including dry matter, crude protein, ether extract (crude lipid), crude fiber, ash, nitrogen-free extract (NFE), and gross energy was determined in duplicate following the standard regulatory methods of the Association of Official Analytical Chemists (AOAC) [30].

For mineral profiling, dried diet samples were finely ground and subjected to wet acid digestion using a nitric acid–hydrochloric acid mixture (1:3 *v*/*v*) at 200 °C for 30 min in accordance with the Korean Food Standard Codex [31,32]. Digested solutions were cleared through 0.45 µm Whatman membrane filters, stored in acid-washed glass vials, and quantified via Inductively Coupled Plasma–Optical Emission Spectrometry (ICP-OES; 720 series, Agilent Technologies, Santa Clara, CA, USA).

### 2.4. Amino Acid and Fatty Acid Profiling

Amino acid profiles were resolved on a freeze-dried basis. Samples were subjected to total acid hydrolysis with 6 N HCl at 110 °C for 24 h under a protective nitrogen atmosphere. Reconstituted hydrolysates (reconstituted in a 0.12 N physiological citrate buffer, pH 2.20) were analyzed using an automated amino acid analyzer (Sykam S633; Sykam GmbH, Bayern, Germany) equipped with a Sykam LCA L-07 categorical ion-exchange column according to established protocols [32].

Fatty acid profiles were determined by extracting total lipids and converting the fatty acid fractions into fatty acid methyl esters (FAMEs) via transesterification [33]. FAME profiling was performed using gas chromatography equipped with a flame ionization detector (GC-FID; GC-14B, Shimadzu, Japan) using an SP-2560 capillary column with nitrogen as the carrier gas (300 kPa) in accordance with the Korean Food Standard Codex [31]. The column oven temperature profile was programmed from 170 °C to 205 °C at a ramp rate of 1 °C/min with a split ratio of 100:1. Fatty acid compositions were identified by retention-time matching against certified reference standards (Sigma-Aldrich, St. Louis, MO, USA) and expressed as relative percentages of the total lipid fraction.

### 2.5. Honey Bee Physiological Performance and Longevity

#### 2.5.1. Hypopharyngeal Gland (HPG) Acini Development

On day 7 of dietary exposure, five worker bees from each treatment were randomly sampled to assess HPG acini development. The glands were carefully dissected and immersed in droplets of physiological saline solution (0.85% of NaCl) in plastic Petri dishes maintained at 25 °C. Digital micrographs were captured by a stereo microscope camera (Leica Microsystems Fluorescence Stereo Microscopes Leica M205 FCA and Leica M205 FA, Wetzlar, Germany) and a digital microscope camera (Hirox HRX-01 and RX-100, Oreland, NJ, USA) [17]. For each individual HPG, the diameters of 20 randomly selected HPG acini with well-defined cellular borders were measured using ImageJ analysis software (version 1.53k, National Institutes of Health, Bethesda, MD, USA). The average values were analyzed per diet, and mean values were used for subsequent statistical analyses. 

#### 2.5.2. Laboratory Longevity Bioassays

Honey bee colonies were maintained under uniform apiary conditions at the Department of Entomology and Plant Pathology, Faculty of Agriculture, Chiang Mai University, Thailand (19°04′21.432″ N, 99°53′03.6234″ E) from October to November 2025 [34]. Sealed brood frames were pulled from three healthy, independent donor colonies and placed into an insect growth incubator under dark conditions (33 ± 1.0 °C, 60 ± 1.0% RH) until adult worker emergence.

Cohorts of 30 newly emerged worker bees from a mixed colony were pooled and transferred into 600 mL ventilated polypropylene cages (40 ventilation ports, ≤5 mm diameter). For each of the six dietary treatments, three independent cages were utilized as biological replicates (totaling 90 bees monitored per treatment). Cages were equipped with gravity-fed syringe reservoirs containing pure water and a 50% sucrose syrup solution. Solid experimental diet patties (MP, SB, SBM, SBW, SBB) were offered ad libitum at 2 g per cage via specialized plastic feeders. Feed stocks and liquid syringes were completely refreshed at 3-day intervals. Survival and mortality rates were documented daily across a 35-day experimental window, and dead bees were extracted immediately upon verification.

### 2.6. Gut Microbiota Metagenomic Sequencing

Metagenomic analysis was conducted on genomic DNA extracted from honey bee hindgut samples to preserve native microbial community composition, following the laboratory protocols described by Praphawilai et al. [17]. Hindguts were dissected and pooled to obtain sufficient biomass for DNA extraction. Because gut size and content varied among individuals, the number of hindguts per pooled sample ranged from 5 to 10. To capture true biological variation while ensuring uniform starting material, individuals were pooled to form three independent biological replicates per treatment group, with each distinct biological replicate subsequently processed in technical triplicate during extraction. Approximately 250 mg of each pooled sample was processed using either the DNeasy PowerSoil^®^ Pro Kit or the DNeasy PowerFecal^®^ Pro Kit (QIAGEN, Hilden, Germany) according to the manufacturer’s protocol. Samples were incubated at 70 °C for 20 min to enhance cell lysis, followed by mechanical disruption via bead beating. DNA integrity was assessed by agarose gel electrophoresis. Purity was evaluated spectrophotometrically using A260/A280 and A260/A230 ratios. DNA concentration was quantified using a Qubit 4 Fluorometer (Thermo Fisher Scientific, Waltham, MA, USA) with the dsDNA High Sensitivity Assay Kit.

The bacterial 16S rRNA gene was amplified using the UltraRun LongRange PCR Kit (QIAGEN, GmbH, Hilden, Germany). Barcoding was incorporated during amplification using the Rapid Sequencing DNA-16S Barcoding Kit 24 V14 (SQK-16S114.24, Oxford Nanopore Technologies, Oxford, UK). Barcoded amplicons were pooled and purified with AMPure XP beads to remove residual primers and PCR byproducts. DNA concentration was also measured using the Qubit Fluorometer (Thermo Fisher Scientific, Waltham, MA, USA) prior to adapter ligation. Sequencing adapters and buffers were added based on the manufacturer’s instructions.

Sequencing was performed on a MinION Mk1D device (Oxford Nanopore Technologies, Oxford, UK). Flow cells were equilibrated to room temperature before loading, and sequencing runs were monitored using MinKNOW™ software (version 25.05.14, Oxford Nanopore Technologies plc, Oxford, UK). Raw signal data were base-called to generate FASTQ files. A total of 21 pooled gut samples yielded barcode-assigned reads and were included in downstream analysis. Sequencing depth ranged from 20,467 to 37,178 reads per sample (mean: ~25,687 reads), with 539,424 total reads across all samples. Taxonomic profiling was conducted using the EPI2ME 16S workflow for genus-level classification and the EPI2ME metagenomics workflow for species-level identification and relative abundance estimation (EPI2ME release 25.12; Oxford Nanopore Technologies plc, Oxford, UK). Downstream microbial community analyses, including alpha diversity, beta diversity ordination (PCoA), and statistical comparisons, were performed using MicrobiomeAnalyst 2.0.

### 2.7. Statistical Analyses

Data that met the assumptions of normality were analyzed using one-way analysis of variance (ANOVA), followed by Tukey’s post hoc test to compare means among diets. Statistical significance was set at *p* < 0.05. All univariate statistical analyses were performed using SPSS software (version 27.0; IBM Corp., Armonk, NY, USA). Honey bee survival data were evaluated using the Kaplan–Meier estimator to construct survival curves for each dietary treatment, with median survival time defined as the time at which cumulative survival first fell to ≤50%; diets in which cumulative survival remained above 50% through the end of the 35-day observation period were designated as median survival “not reached.” Differences in survival among diets were assessed using the log-rank (Mantel–Cox) test, both globally across all six treatments and via pairwise post hoc comparisons between individual diets, using the same SPSS software and significance threshold. Because bees within the same cage may not be fully independent, survival comparisons were additionally verified using an exact permutation test at the level of replicate cages: for each pairwise diet comparison, the three replicate cages per diet were reassigned to the two diet labels in all C (6,3) = 20 possible ways, and the log-rank statistic was recalculated for each reassignment using the full bee-level survival data. This approach treats cage as the unit of exchangeability while retaining the timing information in the survival curves; the smallest attainable exact *p*-value under this test is 0.05 for a one-sided comparison, or approximately 0.10 in practice given the symmetry of two-group reassignments. Multivariate relationships among diets and response variables were examined using principal component analysis (PCA). Prior to performing the PCA, data standardization was applied to ensure data consistency. An orthogonal transformation was applied following the method described by Brereton [35]. Computations of principal component scores and graphical outputs were generated using JMP software (version 10; SAS Institute Inc., Cary, NC, USA). Results were visualized as two-dimensional biplots displaying principal component scores and loadings, with emphasis on the first two principal components (PC1 and PC2), facilitating interpretation of patterns and associations among diets [36].

## 3. Results

### 3.1. Proximate and Mineral Composition of Experimental Diets

The proximate composition of the experimental diets differed significantly among different diets (*p* < 0.05 for all parameters). Proximate nutrient analysis showed significant differences between the MP diet and all seabass-based diets, irrespective of insect supplementation (Table 1). The MP diet exceeded all seabass-based diets in every nutrient measured: dry matter content (75.64% vs. 57.66–63.57%), crude protein (17.24% vs. 12.48–15.59%), ether extract (8.92% vs. 3.10–5.62%), crude fiber (5.35% vs. 2.18–2.24%), nitrogen-free extract (47.40% vs. 38.69–43.98%), and metabolizable energy (341.88 vs. 250.61–265.21 kcal/100 g). Consistent with the proximate composition, the MP diet also exhibited higher mineral content, except for sodium, than seabass-based diets (Table 2). Among microminerals, calcium (1850.69 mg/kg) and phosphorus (4606.31 mg/kg) in MP exceeded those in SB (Ca 848.62 mg/kg; P 2348.29 mg/kg) and insect-supplemented diets (Ca 539.17–895.45 mg/kg; P 2117.93–2572.74 mg/kg). Potassium (2157.55 mg/kg) and magnesium (1097.76 mg/kg) were similarly enriched, whereas sodium was lowest in MP (125.13 mg/kg) compared with seabass diets (~1500 mg/kg). Trace elements followed the same pattern: manganese (22.58 mg/kg), iron (99.35 mg/kg), and copper (6.75 mg/kg) were higher than in seabass-based diets (Mn 4.80–8.39 mg/kg; Fe 19.22–30.49 mg/kg; Cu 1.51–3.43 mg/kg). Arsenic levels were low and comparable across all diets (0.32–0.61 mg/kg, *p* = 0.393), indicating no contamination risk.

### 3.2. Amino Acid and Fatty Acid Composition of Experimental Diets

#### 3.2.1. Amino Acid Composition

**Figure 1:**
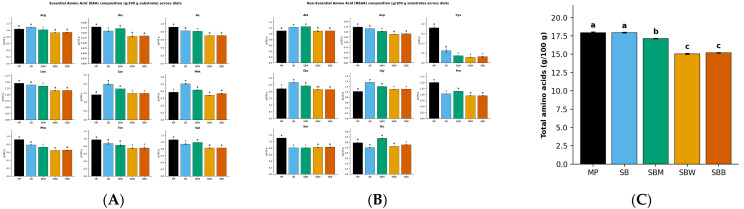
Amino acid composition (g/100 g substrate) of the five experimental diets. (**A**) Essential amino acid (EAA) content; (**B**) non-essential amino acid (NEAA) content; (**C**) total amino acid content. Bars represent mean ± SD (n = 3 per diet). Diets are color-coded as follows: black = MP (mixed pollen diet); blue = SB (seabass-based diet); teal = SBM (sea-bass–mealworm-based diet); yellow = SBW (seabass–wax moth-based diet); orange = SBB (seabass–black soldier fly-based diet). Different lowercase letters above bars indicate significant differences among diets (Tukey’s post hoc test, *p* < 0.05); bars sharing the same letter are not significantly different.

**Figure 2:**
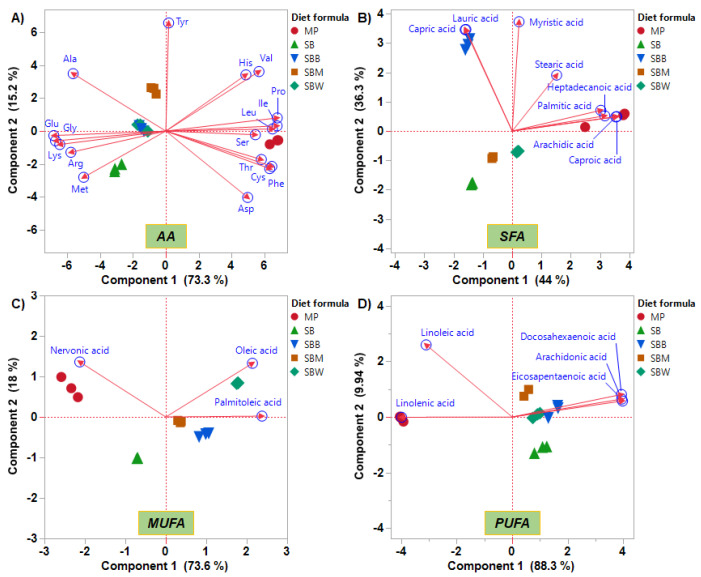
Principal component analysis (PCA) of amino acids; AA (**A**), saturated fatty acid; SFA (**B**), monounsaturated fatty acid; MUFA (**C**) and polyunsaturated fatty acid; PUFA (**D**) in five diet formulas; MP = mixed pollen diet, SB = seabass-based diet, SBM = seabass–mealworm-based diet, SBW = seabass–wax moth-based diet and SBB = seabass–black soldier fly-based diet.

The amino acid profiles revealed distinct differences among diets, particularly in essential amino acids (EAAs) crucial for honey bee growth and physiology. The MP diet exhibited a well-balanced EAA composition, with high levels of leucine, isoleucine, valine, phenylalanine, and threonine (Figure 1). In contrast, seabass-based diets were characterized by selectively elevated lysine and methionine contents. The individual amino acid levels, and thus total amino acid content, mirrored the crude protein content. PCA of amino acid profiles clearly distinguished the MP diet from the others along the PC1 axis (variance explanation of 73.3%), while seabass-based diets were slightly distributed along the secondary component (variance explanation of 15.2%) (Figure 2A). The PC loadings suggested that the MP diet positively correlated with EAAs, resembling natural pollen, while seabass-based diets associated with lysine, methionine, arginine, and glutamic acid. Insect-supplemented diets (SBM, SBW, and SBB) showed intermediate profiles, and the loadings pointed out non-essential amino acids (NEAAs), notably tyrosine and alanine, as differentiators.

#### 3.2.2. Fatty Acid Composition

Fatty acid composition varied significantly among diets (*p* < 0.001), particularly for saturated (SFAs) and polyunsaturated fatty acids (PUFAs) (Table 3). The MP diet was characterized by high concentrations of plant-derived PUFAs, particularly α-linolenic acid (omega-3; 1434.66 mg/100 g) and linoleic acid (omega-6; 355.43 mg/100 g), which were much lower in seabass-based diets. Conversely, omega-3 fatty acids DHA and EPA were exclusive to seabass-based diets, highest in SBB, and absent in MP. Saturated fatty acid profiles also varied substantially among diets. Palmitic acid dominated all diets, with particularly high levels in the MP (780.61 mg/100 g) and SBW (752.44 mg/100 g). Lauric acid was very high in SBB (604.30 mg/100 g) but was absent in the other diets.

#### 3.2.3. Principal Component Analysis of Nutritional Composition

The PCA of SFAs clearly separated diets along the primary component (Figure 2B). MP was associated with palmitic, arachidic, caproic, heptadecanoic, and stearic acids, while seabass-based diets, especially SBB, were highly associated with medium- and long-chain SFAs. For monounsaturated fatty acids (MUFAs), PC1 distinguished insect-supplemented diets (SBM, SBW, SBB) from MP and SB, mainly due to oleic and palmitoleic acids, with SBW and SBB more strongly associated with MUFA vectors (Figure 2C). The PCA of PUFAs showed pronounced diet discrimination (Figure 2D), where MP clustered clearly in PC space with plant-derived PUFAs. The loadings, on the other hand, suggested that seabass diets tended to be associated with marine long-chain PUFAs (EPA, DHA).

**Figure 3:**
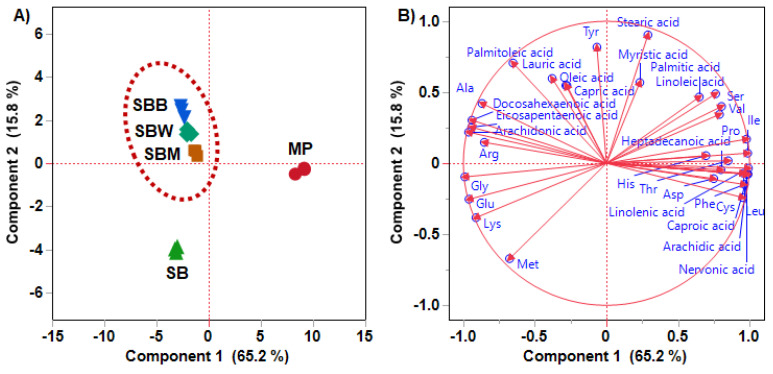
Principal component scores (**A**) and loadings (**B**) of all amino and fatty acids from five diet formulas; MP = mixed pollen diet, SB = seabass-based diet, SBM = seabass–mealworm-based diet, SBW = seabass–wax moth-based diet and SBB = seabass–black soldier fly-based diet.

The PCA integrating all amino acids and fatty acids revealed a clear separation among diets in the score plot (Figure 3A), indicating distinct overall nutrient compositions among the five diet formulations. The MP diet formed a distinct cluster, separated from the seabass-only (SB) diet along the primary component (65.2% variance explained), while the insect-supplemented diets (SBM, SBW, and SBB) occupied intermediate positions between MP and SB. The loading plot indicated that this separation was driven by contrasting contributions of amino acids and fatty acids (Figure 3B). The MP diet was positively associated with certain essential amino acids and plant-derived PUFAs, whereas the SB diet showed stronger associations with seabass-derived amino acids and long-chain marine PUFAs. The remaining three insect-supplemented diets exhibited mixed loadings, reflecting partial convergence of both amino acid and fatty acid profiles relative to the MP and SB diets.

### 3.3. Physiological Responses and Survival of Honey Bees

#### 3.3.1. Hypopharyngeal Gland Acini Development

**Figure 4:**
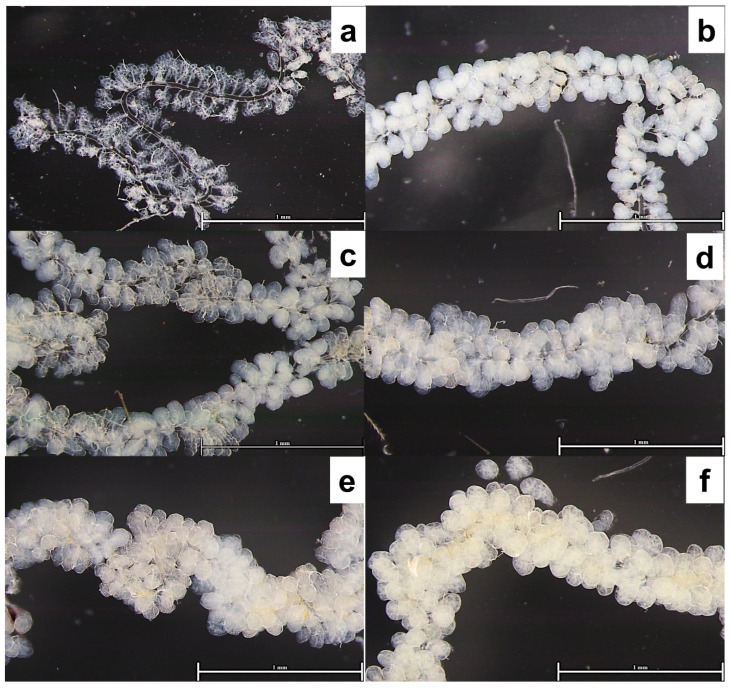
HPG acini of honey bees fed artificial pollen diets from marine fish and insect larvae; SR = syrup without diet (**a**), MP = mixed pollen diet (**b**), SB = seabass-based diet (**c**), SBM = seabass–mealworm-based diet (**d**), SBW = seabass–wax moth-based diet (**e**) and SBB = seabass–black soldier fly-based diet (**f**).

Hypopharyngeal gland (HPG) acini width differed significantly among diets (one-way ANOVA, F = 38.51, *p* < 0.0001; *n* = 5 bees/diet, 20 acini averaged per bee) (Figure 4). Honey bees fed syrup without a protein source (SR) exhibited the smallest HPG-acinus width (0.060 ± 0.001 mm), which was significantly lower than that of bees fed every other diet (Tukey’s HSD, all *p* < 0.001). Acinus width increased progressively with dietary protein complexity: the mixed pollen (MP, 0.088 ± 0.001 mm) and seabass-based (SB, 0.091 ± 0.004 mm) diets did not differ significantly from one another (*p* = 0.991), nor did SB differ significantly from the seabass–wax moth diet (SBW, 0.105 ± 0.002 mm; *p* = 0.089). SBW, in turn, did not differ significantly from the seabass–mealworm diet (SBM, 0.115 ± 0.007 mm; *p* = 0.462), and SBM did not differ significantly from the seabass–black soldier fly diet (SBB, 0.123 ± 0.004 mm; *p* = 0.597), which showed the numerically highest acinus width among all diets tested. Non-adjacent diets in this progression differed significantly from one another (e.g., MP vs. SBW, *p* = 0.025; SB vs. SBM, *p* = 0.001; SBW vs. SBB, *p* = 0.022), indicating a graded increase in HPG development with dietary complexity rather than a single diet standing apart from the rest.

#### 3.3.2. Survival of Worker Honey Bees

**Figure 5:**
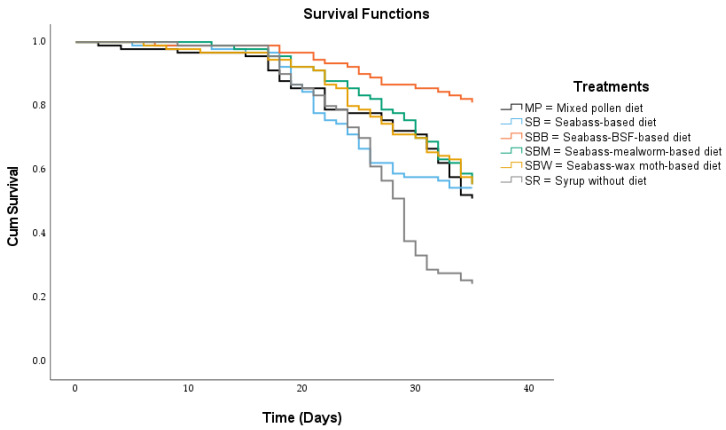
Kaplan–Meier survival curves of adult worker honey bees fed different artificial pollen diets formulated from marine fish and insect larvae; SR = sugar syrup (negative control); MP = mixed pollen diet (positive control); SB = seabass-based diet; SBM = seabass–mealworm-based diet; SBW = seabass–wax moth-based diet; SBB = seabass–black soldier fly-based diet. *n* = 90 bees per diet (three replicate cages of 30 bees each).

Survival of worker bees differed significantly among diets when analyzed at the level of individual bees pooled across replicate cages (log-rank χ^2^ = 61.27, df = 5, *p* < 0.0001) (Figure 5). The SR diet showed a markedly more rapid decline in cumulative survival compared with protein-supplemented diets, particularly after approximately 15–20 days, reaching a median survival time of 29 days and only 24.4% survival by day 35, significantly lower than every other diet (SR vs. MP, χ^2^ = 15.08, *p* < 0.001; SR vs. SB, χ^2^ = 9.92, *p* = 0.002; SR vs. SBM, χ^2^ = 21.83, *p* < 0.0001; SR vs. SBW, χ^2^ = 18.63, *p* < 0.0001; SR vs. SBB, χ^2^ = 58.48, *p* < 0.0001). The MP diet maintained relatively high survival during the early phase of the experiment, ending at 51.1% survival by day 35; median survival was not reached for MP or for any protein-containing diet. Honey bees fed the SB, SBM, and SBW diets showed survival patterns statistically indistinguishable from MP (all pairwise *p* > 0.45), with day-35 survival of 54.4%, 55.6%, and 55.6%, respectively. The SBB diet showed the highest day-35 survival (81.1%) and the most consistent survival across replicate cages. Because bees within the same cage are not fully independent, we additionally verified these comparisons using an exact cage-level permutation test (see Section 2.7). No pairwise comparison reached significance (all exact *p* ≥ 0.10; the floor attainable with this test given three cages per diet, regardless of effect size), including both the SR-vs-diet contrasts and MP vs. SBB. We therefore interpret these results conservatively: SBB showed the highest and most consistent day-35 survival (81.1% ± 5.1%), but all pairwise differences among diets, including the apparent SR decline, should be treated as descriptive trends rather than confirmed effects, pending confirmation with additional replicate cages.

### 3.4. Effects of Diet on the Honey Bee Gut Microbiome

#### 3.4.1. Alpha and Beta Diversity of the Gut Microbiome

**Figure 6:**
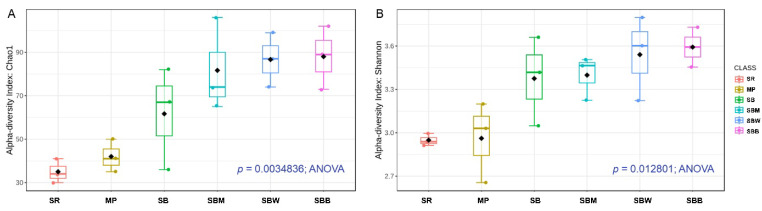
Alpha diversity of the honey bee hindgut microbiome across different dietary treatments (**A**) Chao1 richness index and (**B**) Shannon diversity index calculated from metagenomic sequencing data for bees fed SR, MP, SB, SBM, SBW, and SBB diets. Boxplots represent the median and interquartile range, with whiskers indicating the range of observed values; black diamonds denote group means. Statistical differences among dietary groups were assessed using one-way ANOVA, with corresponding *p*-values shown in each panel; SR = syrup without diet, MP = mixed pollen diet, SB = seabass-based diet, SBM = seabass–mealworm-based diet, SBW = seabass–wax moth-based diet and SBB = seabass–black soldier fly-based diet.

Alpha diversity of the hindgut bacterial communities was evaluated using the Chao1 richness estimator and the Shannon diversity index (Figure 6). Significant differences in alpha diversity were observed among dietary groups for both metrics. The Chao1 index, reflecting estimated species richness, differed significantly across diets (*p* = 0.0034836, *F* = 6.642). Honey bees fed syrup only (SR) exhibited the lowest richness, followed by the pollen-based diet (MP). In contrast, all seabass-based diets showed markedly higher richness. Among the diets, those with insect larvae (SBM, SBW, and SBB) displayed the highest Chao1 values, indicating a substantially expanded bacterial repertoire in the hindgut. However, the SB diet showed intermediate richness higher than that of SR and MP but lower than that of insect-based diets.

Consistent patterns were observed for the Shannon index, which accounts for both richness and evenness. Shannon diversity differed significantly among diets (*F* = 4.7315, *p* = 0.0128). The SR diet again exhibited the lowest diversity, whereas the MP diet exhibited a modest increase. The SBW and SBB diets supported the highest Shannon indices, indicating not only greater richness but also a more even distribution of bacterial taxa. In contrast, the MW and SB groups displayed intermediate Shannon diversity values.

**Figure 7:**
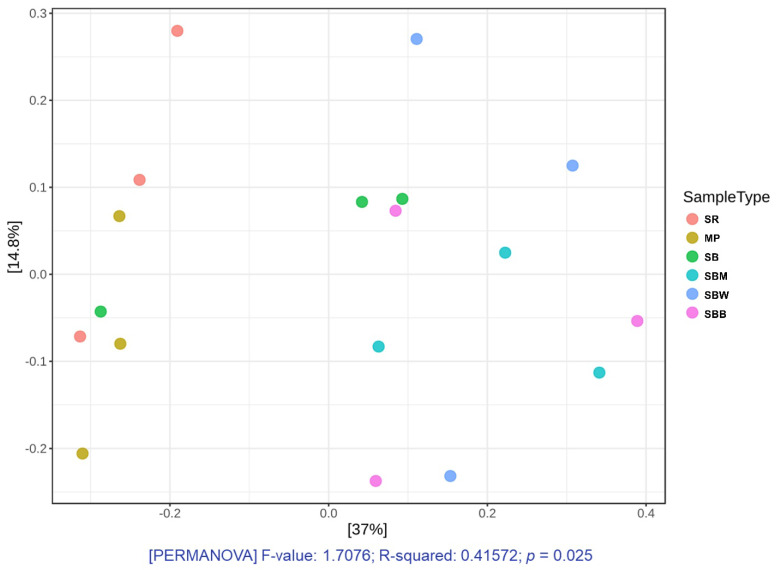
Principal coordinate analysis (PCoA) of hindgut bacterial communities across diets. Percentages on axes indicate the proportion of variance explained. PERMANOVA statistics are shown above the plot; SR = syrup without diet, MP = mixed pollen diet, SB = seabass-based diet, SBM = seabass–mealworm-based diet, SBW = seabass–wax moth-based diet and SBB = seabass–black soldier fly-based diet.

Beta diversity analysis was performed to assess differences in hindgut bacterial community composition among diets. Principal coordinate analysis (PCoA) based on a distance matrix revealed clear separation of samples according to diet (Figure 7). The first two principal coordinates explained 37.0% (PC1) and 14.8% (PC2) of the total variation in community composition. Permutational multivariate analysis of variance (PERMANOVA) confirmed that experimental diets significantly influenced hindgut microbial structure (PERMANOVA, *F* = 1.7076, *R*^2^ = 0.41572, *p* = 0.025), indicating that approximately 41.6% of the variation in bacterial community composition could be attributed to diet. Samples from the SR clustered distinctly from those receiving pollen or seabass-based diets, indicating a simplified and compositionally distinct microbiome under syrup feeding. The MP diet formed a partially overlapping but separable cluster, suggesting a moderate shift in microbial structure relative to SR (syrup alone). In contrast, seabass-based diets exhibited greater dispersion and partial separation along PC1, reflecting diet-specific microbial configurations. Among the diets, those with insect larvae (SBM, SBW and SBB) tended to cluster further from the SR and MP diets, indicating more pronounced alterations in community composition. The SB diet occupied an intermediate position, overlapping partially with insect-based diets while remaining distinct from the SR diet.

#### 3.4.2. Taxonomic Composition of the Gut Microbiome

**Figure 8:**
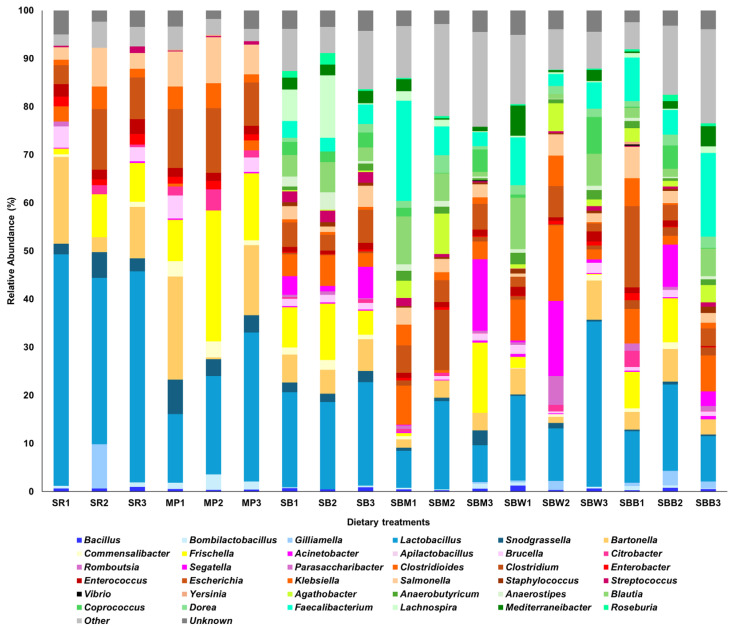
Relative abundance of bacterial communities at the genus level across diets. Stacked bar plots illustrate the taxonomic composition of microbial communities classified at the genus ranks; SR = syrup without diet, MP = mixed pollen diet, SB = seabass-based diet, SBM = seabass–mealworm-based diet, SBW = seabass–wax moth-based diet and SBB = seabass–black soldier fly-based diet.

The composition and persistence of the hindgut core microbiome varied markedly among diets when evaluated across increasing detection thresholds of relative abundance (Figure 8). Core gut bacteria were present across all diets but showed pronounced diet-dependent restructuring. The SR diet was strongly dominated by core bacteria of the genus *Lactobacillus*, which accounted for the majority of the community, accompanied by moderate abundances of *Snodgrassella* and low levels of *Bombilactobacillus*. In contrast, among protein-containing diets, reduced dominance of core bacteria was observed. However, only SB diets showed a relative increase in short-chain fatty acid (SCFA)- associated bacteria, including genera commonly associated with fermentative or anaerobic metabolism such as *Faecalibacterium*, *Blautia*, *Dorea*, *Anaerobutyricum*, *Agathobacter*, and *Roseburia*. Several of these genera appeared sporadically or at moderate abundance across seabass-based (SB) diet-fed groups, contributing to greater microbial heterogeneity compared with carbohydrate (SR) and pollen-based diets (MP). Putative pathogen-related genera (*Enterococcus*, *Escherichia*, *Klebsiella*, *Salmonella*, *Streptococcus*, and *Staphylococcus*) were detected across all diets.

**Figure 9:**
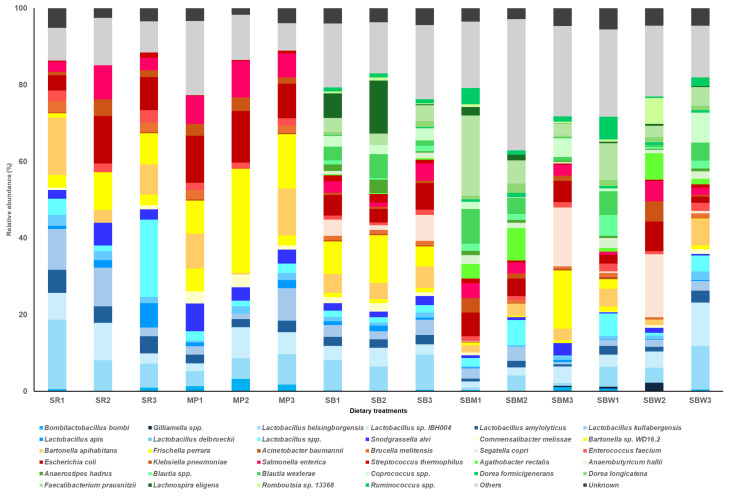
Relative abundance of bacterial communities at the species rank across diets. Stacked bar plots illustrate the taxonomic composition of microbial communities classified at the species rank; SR = syrup without diet, MP = mixed pollen diet, SB = seabass-based diet, SBM = seabass–mealworm-based diet and SBW = seabass–wax moth-based diet.

At the species level, the gut microbiota of honey bees could be clearly categorized into core, non-core, and transient bacterial groups, with distinct patterns observed among diets (Figure 9). The syrup (SR) and pollen-based (MP) diets were dominated by core gut symbionts of *Lactobaclillu* spp. such as *L. helsingborgensis* (*Lactobacillus* Firm-5), *L. kullabergensis*, and *Lactobacillus* sp. IBH004. In contrast, SCFA-associated bacteria were not detected in the SR and MP diets but were observed in honey bees receiving seabass-based diets, where they likely occurred as transient taxa associated with dietary exposure. These bacterial taxa, including *Faecalibacterium prausnitzii*, *Blautia wexlerae*, *Dorea longicatena*, *Anaerobutyricum hallii*, *Agathobacter rectalis*, and *Romboutsia* sp. 13368, have not been previously reported in studies of the honey bee gut microbiome based on the currently available literature. Transient taxa were primarily represented by environmental and opportunistic bacteria such as *Enterococcus faecium*, *Escherichia coli*, *Klebsiella pneumoniae Salmonella enterica* and *Streptococcus thermophilus*.

#### 3.4.3. Linear Discriminant Analysis Effect Size and Random Forest Analysis of Microbial Biomarkers

**Figure 10:**
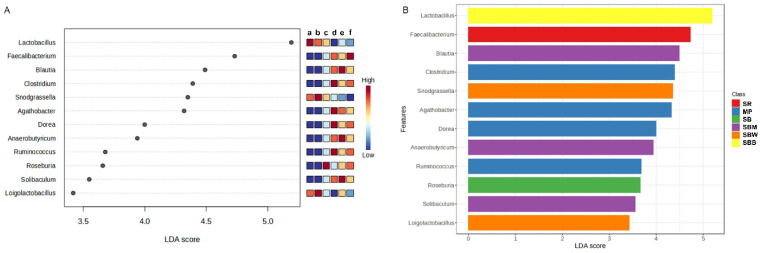
LEfSe analysis identifying differentially abundant bacterial genera among diets. Linear discriminant analysis effect size (LEfSe) was applied at the genus level to identify taxa that differed significantly among dietary groups using an original Kruskal–Wallis test *p*-value cutoff of 0.05. (**A**) LDA scores of discriminative genera, with higher scores indicating stronger effect sizes. Colored heatmap bars indicate relative abundance patterns of each genus across diets; letters a–f correspond to the six dietary groups (a = SR; syrup without diet, b = MP; mixed pollen diet, c = SB; seabass-based diet, d = SBM; seabass–mealworm-based diet, e = SBW; seabass–wax moth-based diet and f = SBB; seabass–black soldier fly-based diet). (**B**) Bar plot showing LDA scores of the same genera, colored by the dietary group in which each taxon was most strongly enriched. Only genera meeting both the statistical significance threshold (*p* < 0.05) and the LDA effect size criterion are shown.

Linear discriminant analysis effect size (LEfSe) identified multiple bacterial genera that were differentially enriched among diets based on both statistical significance and effect size (Figure 10). At the genus level, *Lactobacillus* exhibited the highest LDA score and was strongly associated with the SR diet, reflecting the dominance of carbohydrate- utilizing bacteria. *Snodgrassella* and *Loigolactobacillus* were enriched in the MP diet, consistent with the persistence of canonical honey bee gut symbionts under plant-derived nutrition. In contrast, SB diets were characterized by enrichment of a broader set of discriminatory SCFA-associated bacteria, including *Faecalibacterium*, *Blautia*, *Agathobacter*, *Anaerobutyricum*, *Dorea*, and *Ruminococcus*, all of which exhibited moderate to high LDA scores. Together, these taxa contributed strongly to discrimination among dietary groups, indicating that SB diets drive pronounced and diet-specific shifts in gut microbial composition, supporting increased microbial heterogeneity relative to SR (syrup) and MP (pollen-based) diets.

## 4. Discussion

### 4.1. Nutritional Composition Relative to Natural Pollen

The nutritional profiles observed among the experimental diets highlight the inherent differences between natural pollen and formulated substitutes. In managed honey bee colonies used for pollination services, adequate and balanced nutrition is essential for maintaining colony strength, worker longevity, and overall colony performance [1,2,9]. Natural pollen is widely recognized as a nutritionally balanced feed source for honey bees, providing diverse proteins, lipids, carbohydrates, and micronutrients required for normal physiological functions [10,11]. The comparatively richer nutrient composition of the mixed pollen diet therefore reinforces its role as a biological reference for evaluating the nutritional adequacy of alternative dietary formulations or artificial pollen diets designed to support colony health during periods of limited pollen availability [20,21]. Marine fish proteins are known to provide a broad spectrum of essential amino acids and digestible nutrients [27]. Recent studies have explored the use of marine fish hydrolysates incorporated into syrup-based diets as a strategy to enhance the nutritional profile and improve nutrient availability for honey bees, which may help maintain physiological performance in adult worker bees [28]. Although the SB diets exhibited lower overall nutrient content than the MP diet, the presence of animal-derived proteins and lipids suggests nutritional potential comparable to several artificial pollen diets that primarily rely on plant-based ingredients [15,21,34]. Furthermore, these values align with several commercial products that have demonstrated effectiveness at protein levels comparable to those observed in the present study, including Global (16.6%), Bulk Soft (16.1%), Healthy Bees (15.3%), and AP23 (14.7%). In contrast, other commercial formulations contain higher protein concentrations, such as Ultra Bee (22.0%), Mega Bee (21.6%), and Homebrew (20.9%) [15]. Moreover, the lipid content (ether extract) in our formulations was comparable to that of commercial diets, which typically contain approximately 1–5% fat [15].

However, the MP diet exhibited a relatively higher lipid level (8%), likely due to the inclusion of natural pollen as a primary component, as many pollen types are known to contain elevated lipid contents [24]. Relative to natural pollen (MP), the seabass-based diets contained 9–28% less crude protein (12.48–15.59% vs. 17.24%), less than half the ether extract, and lower calcium, phosphorus, and potassium, but uniquely supplied marine long-chain PUFAs (EPA, DHA) and, in SBB, lauric acid. Components are entirely absent from natural pollen.

Minerals also represent an important component of honey bee nutrition because they participate in metabolic regulation, enzyme activity, and cellular homeostasis [37]. As a mineral and vitamin premix specifically formulated for honey bees was not available in Thailand, a livestock-grade premix was used instead. The MP and SB diets exhibited broadly similar overall mineral profiles, comprising both macro- and trace elements. However, the SB diet contained a wider range of minerals at lower concentrations and was characterized by a notably higher sodium content compared to the MP diet. Therefore, the formulation may require the inclusion of a combined mineral–vitamin premix at an increased level to better match the nutritional profile of the MP diet, which represents mixed pollen, and to more closely approximate the composition of bee pollen and bee bread [38]. In addition, the incorporation of insect-derived ingredients may enhance the biochemical diversity of the diet by introducing additional protein, lipid, and micronutrients that are naturally present in insect tissues [39] and may partially resemble components found in pollen.

Differences in amino acid profiles highlight the critical importance of amino acids requirement in honey bee nutrition [19]. A well-balanced amino acid composition rather than total protein content alone is fundamental to maintaining bee health and performance [40,41]. A balanced amino acid composition of a feed source is a key determinant of pollinator health and is more important than total protein content. It supports physiological functions and contributes to increased longevity of worker bees, thereby enhancing their capacity for effective foraging and pollination [42]. The MP diet, which exhibited a well-balanced essential amino acid (EAA) profile comparable to natural pollen [24,43], is therefore more likely to support optimal health, longevity, and foraging efficiency in honey bees than the SB diet. In contrast, the SB diet contained higher levels of certain EAAs important for bees, such as lysine and methionine [44]. This indicates that the inclusion of seabass and insect-derived proteins can enhance specific essential amino acids in the formulation, even though the overall amino acid profile may remain less balanced than that of natural pollen-based diets (MP diet).

The presence of these lipid components, particularly seabass-derived omega-3 fatty acids such as DHA and EPA [45] and lauric acid from BSF [46] in combination, represents a distinctive feature of the formulated diets not reported in single-ingredient formulations. This contrasts with the MP diet, which closely mirrors the fatty acid composition of natural pollen and includes only one omega-3 fatty acid, linoleic acid (ALA) [24]. In contrast, the composition of SFAs differed among diets. Palmitic acid was predominant across all diets, while lauric acid was uniquely abundant in SBB, likely due to the inclusion of BSF, which is rich in this fatty acid [46]. This differs from previous studies, which indicate that honey bees primarily obtain omega-3 fatty acids from pollen in the form of ALA and do not acquire lauric acid from seabass (SB) or BSF-supplemented (SBB) diets [47].

### 4.2. Physiological Development: Hypopharyngeal Glands and Survival

The hypopharyngeal glands (HPGs) of worker bees are responsible for producing royal jelly, a process that depends heavily on adequate pollen reserves to stimulate gland activity. Consequently, the availability and quality of natural forage during the beekeeping season can strongly influence both the quantity and nutritional quality of the royal jelly produced [48]. The differences in HPG acini development among diets align with previous findings that high-quality artificial pollen diets promote greater gland development than pollen-free or nutrient-limited diets [22]. In the present study, honey bees fed seabass-based diets (SB series) exhibited significantly larger HPG acini than those receiving the pollen-free treatment (SR). These results support earlier findings that protein-based diets and commercial products can stimulate HPG development in a manner comparable to natural bee bread [15,49].

Honey bees fed the SBB and SBM diets exhibited the greatest HPG acini development among all diets tested (0.123 mm and 0.115 mm, respectively; Tukey’s HSD, *p* < 0.05 vs. all other diets), though SBB and SBM were not statistically distinguishable from one another (*p* = 0.597). This is a notable result considering the crude protein content of both diets (13.25% and 14.05%, respectively) was statistically lower than that of the natural mixed pollen positive control (17.24%). This finding is consistent with our hypothesis that the nutritional quality, bioavailability, and digestibility of an enzymatically hydrolyzed animal protein complex rather than raw protein volume alone contributes to physiological development, though this derives from a modest sample (*n* = 5 bees/diet) and warrants confirmation with larger cohorts. Processing marine and insect biomass with acid protease likely bypassed the mechanical and enzymatic constraints adult bees face when digesting intact plant-derived protein bodies or pollen walls, leading to rapid systemic amino acid utilization. Furthermore, this exceptional glandular growth was uniquely accelerated by the structural benefits of black soldier fly larvae biomass, which provided a massive reserve of lauric acid (604.30 mg/100 g) absent in all other formulations. Rather than acting as a dietary deterrent or having an uncertain impact, this novel lipid–protein matrix exerted a clear synergistic effect, proving that structurally targeted, highly digestible animal-derived nutrient complexes can functionally supplant traditional raw protein metrics to mitigate nutritional stress without causing detrimental effects to HPG development.

This physiological advantage translated directly into survival outcomes. Honey bees consuming protein-containing diets (MP and all SB diets) exhibited significantly longer lifespans compared with those fed syrup alone (SR), emphasizing the fundamental importance of adequate protein intake in supporting longevity [34]. In particular, honey bees fed the SBB diet lived significantly longer compared with those receiving all other formulations, indicating that the synergistic effects of the hydrolyzed protein and novel lipid components extend beyond glandular health to actively enhance honey bee survival. The successful inclusion of mealworm in the SBM diet and BSF in the SBB diet demonstrates the viability of these alternative matrixes, which aligns with their growing adoption as highly digestible protein sources across various animal feed sectors [50].

However, their application in combination within a single artificial pollen formulation, rather than as standalone protein source, represents the novel element of this approach. Specifically, the incorporation of wax moth larvae in the SBW diet was well accepted by the honey bees, supporting health, and longevity. This outcome is particularly striking given that wax moths are natural pests of honey bees [51], highlighting an unexpected tolerance and potential for utilizing unconventional insect protein sources in artificial pollen diets. Therefore, to ensure that honey bees maintain good health and forage effectively, the use of artificial pollen diets containing animal-derived proteins, as exemplified in the present study with marine fish or combined with insect protein, may serve as an effective strategy to support colony health and enhance pollination performance.

### 4.3. Gut Microbiome Shifts and Novel Fermentative Taxa

Microbiota composition in honey bees is crucial, as it is influenced by diet and plays a key role in reproductive development [52]. Syrup-only (SR) treatment exhibited the lowest alpha diversity, indicating a simplified gut community under protein- and nutrient-limited conditions. The pollen-based (MP) diet moderately increased richness and evenness, reflecting the nutritional contribution of natural pollen. Seabass-based diets generally enhanced microbial diversity, with the highest alpha diversity observed in insect-supplemented diets (SBM, SBW, SBB), suggesting that combining fish- and insect-derived proteins provides substrates that support a broader and more balanced bacterial community. The SB diet showed intermediate diversity, higher than SR and MP but lower than insect-containing diets. Beta diversity analysis revealed clear separation of samples by diet. SR samples clustered distinctly, while MP samples shifted moderately. Seabass-based diets, particularly those with insect protein, clustered further apart, highlighting pronounced diet-driven changes in microbial composition. These results are consistent with previous research on the use of various artificial pollen diets, which found that changes in diet formulation affect the honey bee gut microbiome [52].

The gut microbiota of honey bees serves as a useful model for host-microbe studies due to its low complexity and similarity to mammalian systems [13]. It is primarily composed of a small group of core bacteria including five key genera—*Lactobacillus*, *Bombilactobacillus*, *Gilliamella*, *Snodgrassella*, and *Bifidobacterium*—along with several frequently occurring non-core bacteria such as *Frischella*, *Bartonella*, and *Commensalibacter*. Collectively, these groups account for more than 90% of the gut microbial diversity in honey bees [14,52]. Although the core gut microbiota of honey bee workers is generally stable, individual bees show variation in the relative abundance of core species and in the presence of transient or opportunistic bacteria [16].

In this study, the proportion of core and non-core bacteria observed in this study was lower, accounting for only approximately 50–70%, in contrast to higher proportions reported in earlier studies [52]. Honey bees fed SR and other diets possessed all core gut microbiota taxa, including *Lactobacillus*, *Bombilactobacillus*, *Snodgrassella*, and *Gilliamella*, and exhibited a high diversity of *Lactobacillus* species. These results align with previous studies on the honey bee gut microbiome, which consistently report *Lactobacillus* as a dominant bacterial taxon. [53,54]. In addition to core and non-core bacteria, environmental and pathogen-related bacterial groups were also detected in the honey bee gut microbiome, particularly in samples from the SR and MP diets. However, these groups were present at lower abundances compared to the core and non-core bacterial communities [55].

However, a novel finding of this study is that SB diets resulted in a marked increase in SCFA-associated bacteria across all samples, with prominent taxa including *Faecalibacterium prausnitzii*, *Blautia wexlerae*, *Coprococcus* spp., *Lachnospira eligens*, and *Anaerostipes hadrus*. These bacterial groups are commonly reported to be associated with the fermentation of indigestible carbohydrates and oligosaccharides, which results in the production of short-chain fatty acids (SCFAs), including butyrate, propionate, and acetate, and are often represented by genera such as *Roseburia*, *Bifidobacterium*, and *Faecalibacterium* [56]. However, the enrichment of SCFA-associated bacteria in the honey bee gut remains largely unexplored, suggesting a potentially novel SB diet–microbiome interaction. Therefore, the enrichment of SCFA-associated bacteria may enhance host energy metabolism, gut health, and physiological resilience, potentially improving the foraging efficiency and overall fitness of honey bees, thereby supporting their pollination services. Collectively, these findings substantiate the novelty of this study: to our knowledge, this is the first work to link a combined marine fish–insect larvae hydrolysate to concurrent shifts in gut microbiota composition and hypopharyngeal gland development, distinguishing this approach from prior studies that examined fish-derived or insect-derived protein sources in isolation.

## 5. Conclusions

Pollinator services in agroecosystems require reliable nutritional support for managed honey bees, particularly under conditions of floral scarcity. Natural forage alone is often insufficient and unpredictable due to seasonal fluctuations, habitat loss, and agricultural intensification. Therefore, supplemental feeding is essential to maintain colony health, stability, and pollination efficiency. The development of nutritionally balanced artificial pollen diets represents a practical strategy to buffer against resource limitations and ensure consistent pollinator performance. In this context, SB-based diets demonstrate strong potential as alternative pollen sources that can support honey bee health and sustain pollination services. The incorporation of insect larvae further improved dietary performance, highlighting their value as functional ingredients. However, several aspects warrant further investigation, including the specific roles of marine-derived fatty acids (DHA and EPA) from seabass, the functional significance of lauric acid from BSF, and the potential application of other insect resources such as wax moth larvae. A particularly promising direction would be to supplement the SBB formulation with pollen-specific fatty acids identified in this study but absent from our current diets, namely arachidic acid and heptadecanoic acid, and directly benchmark the resulting formulation’s physiological performance, development and survival against the MP diet. Critically, colony-level feeding trials such as ones assessing foraging behavior, comb storage, and hive-product quality, alongside production cost analysis and longer-duration studies, are needed to establish commercial feasibility and confirm the absence of long-term adverse effects. This study was not designed to evaluate toxicological safety; dedicated toxicological assessment, including sublethal and tissue-level endpoints, is a necessary next step before these ingredients can be recommended for practical use. Further investigation of immune and hemolymph parameters is warranted to fully characterize the physiological impact of these diets on honey bee health. These research directions provide valuable opportunities to advance mechanistic understanding and further optimize artificial pollen formulations. Moreover, integrating such diets into beekeeping practices can enhance agroecosystem resilience and support the use of alternative protein sources in the development of artificial pollen diets for long-term pollinator conservation.

## Figures and Tables

**Table 1:** Proximate composition (%) of artificial pollen diets from marine fish and insect larvae.

Parameters	MP	SB	SBM	SBW	SBB	*p*-Value
Dry matter (DM) (%)	75.64 ± 2.02 ^a^	63.57 ± 1.91 ^b^	58.81 ± 1.11 ^c^	57.66 ± 1.45 ^c^	61.58 ± 1.82 ^bc^	<0.001
Ash (%)	1.96 ± 0.13 ^a^	1.12 ± 0.12 ^b^	1.02 ± 0.13 ^b^	1.03 ± 0.08 ^b^	1.05 ± 0.03 ^b^	<0.001
Crude Fiber (CF) (%)	5.35 ± 0.11 ^a^	2.24 ± 0.11 ^b^	2.22 ± 0.11 ^b^	2.19 ± 0.12 ^b^	2.18 ± 0.16 ^b^	<0.001
Ether Extract (EE) (%)	8.92 ± 0.32 ^a^	3.10 ± 0.14 ^d^	4.14 ± 0.86 ^c^	5.62 ± 0.16 ^b^	4.54 ± 0.22 ^c^	<0.001
Crude Protein (CP) (%)	17.24 ± 0.24 ^a^	15.59 ± 0.78 ^b^	13.73 ± 1.09 ^c^	12.48 ± 0.31 ^c^	13.25 ± 0.75 ^c^	<0.001
Nitrogen-Free Extract (NFE) (%)	47.40 ± 1.01 ^a^	43.98 ± 1.13 ^b^	40.85 ± 1.52 ^c^	38.69 ± 0.59 ^d^	42.75 ± 0.46 ^bc^	<0.001
Energy (kcal/100g)	341.88 ± 2.95 ^a^	263.68 ± 14.53 ^b^	250.61 ± 4.97 ^b^	252.54 ± 1.47 ^b^	265.21 ± 2.67 ^b^	<0.001

Note. Data were analyzed using analysis of variance (ANOVA) and are presented as mean ± SD. Means within a column with different superscripts are considered significantly different at *p* < 0.05, based on post hoc multiple comparisons using Tukey’s B test; MP = mixed pollen diet, SB = seabass-based diet, SBM = seabass–mealworm-based diet, SBW = seabass–wax moth-based diet and SBB = seabass–black soldier fly-based diet.

**Table 2:** Mineral content (mg/kg) of diets and controls.

Minerals	MP	SB	SBM	SBW	SBB	*p*-Value
Ca	1850.69 ± 27.67 ^a^	848.62 ± 5.45 ^c^	610.67 ± 5.12 ^d^	539.17 ± 8.43 ^e^	895.45 ± 10.02 ^b^	<0.001
K	2157.55 ± 14.62 ^a^	1645.11 ± 22.01 ^b^	1617.57 ± 8.67 ^b^	1422.24 ± 10.32 ^d^	1566.52 ± 15.54 ^c^	<0.001
Mg	1097.76 ± 39.87 ^a^	433.95 ± 10.335 ^d^	588.38 ± 17.46 ^b^	396.79 ± 14.56 ^d^	519.35 ± 20.19 ^c^	<0.001
Na	125.13 ± 1.91 ^d^	1565.64 ± 8.49 ^a^	1491.10 ± 10.28 ^b^	1358.86 ± 19.35 ^c^	1480.37 ± 34.67 ^b^	<0.001
P	4606.31 ± 58.58 ^a^	2348.29 ± 8.36 ^c^	2572.74 ± 22.77 ^b^	2117.93 ± 30.82 ^e^	2293.57 ± 39.37 ^d^	<0.001
Mn	22.58 ± 0.41 ^a^	4.80 ± 0.02 ^d^	6.13 ± 0.19 ^c^	5.81 ± 0.11 ^c^	8.39 ± 0.20 ^b^	<0.001
Fe	99.35 ± 6.05 ^a^	19.56 ± 8.39 ^b^	22.53 ± 9.01 ^b^	19.22 ± 9.76 ^b^	30.49 ± 17.88 ^b^	<0.001
Cu	6.75 ± 0.53 ^a^	1.51 ± 0.55 ^c^	3.43 ± 0.55 ^b^	2.04 ± 0.59 ^bc^	1.98 ± 0.61 ^bc^	<0.001
As	0.51 ± 0.02	0.61 ± 0.22	0.37 ± 0.25	0.32 ± 0.24	0.50 ± 0.03	0.393

Note. Data were analyzed using analysis of variance (ANOVA) and are presented as mean ± SD. Means within a column with different superscripts are considered significantly different at *p* < 0.05, based on post hoc multiple comparisons using Tukey’s B test. Rows without superscript letters indicate no significant differences; MP = mixed pollen diet, SB = seabass-based diet, SBM = seabass–mealworm-based diet, SBW = seabass–wax moth-based diet and SBB = seabass–black soldier fly-based diet.

**Table 3:** Fatty acid profiles (mg/100 g) of artificial pollen diets from marine fish and insect larvae.

Fatty Acid	MP	SB	SBM	SBW	SBB	*p*-Value
Saturated fatty acid (SFA)						
Arachidic acid	13.47 ± 0.93	0.00	0.00	0.00	0.00	-
Capric acid	0.00	0.00	0.00	0.00	21.23 ± 0.35	-
Caproic acid	4.92 ± 0.05	0.00	0.00	0.00	0.00	-
Heptadecanoic acid	2.52 ± 2.19	0.00	0.00	0.00	0.00	-
Lauric acid	4.69 ± 0.33 ^b^	0.00	0.00	0.00	604.30 ± 7.14 ^a^	<0.001
Myristic acid	82.14 ± 0.74 ^b^	13.30 ± 0.54 ^d^	34.40 ± 1.72 ^c^	17.78 ± 0.54 ^d^	140.50 ± 5.48 ^a^	<0.001
Palmitic acid	780.61 ± 12.73 ^a^	134.77 ± 3.63 ^e^	304.12 ± 9.26 ^d^	752.44 ± 10.37 ^b^	369.12 ± 15.18 ^c^	<0.001
Stearic acid	65.45 ± 6.77 ^ab^	27.74 ± 1.17 ^c^	62.37 ± 0.99 ^b^	73.83 ± 1.32 ^a^	65.70 ± 5.09 ^ab^	<0.001
Monounsaturated fatty acid (MUFA)						
Nervonic acid	35.71 ± 5.38	0.00	0.00	0.00	0.00	-
Oleic acid	85.20 ± 3.95 ^d^	66.76 ± 1.49 ^d^	413.21 ± 11.67 ^b^	772.03 ± 5.23 ^a^	284.93 ± 16.67 ^c^	<0.001
Palmitoleic acid	0.00	11.36 ± 0.22 ^d^	21.43 ± 1.95 ^c^	42.24 ± 0.37 ^b^	48.30 ± 2.69 ^a^	<0.001
Polyunsaturated fatty acid (PUFA)						
Arachidonic acid	0.00	5.93 ± 0.59 ^a^	6.43 ± 0.59 ^a^	6.73 ± 0.40 ^a^	7.14 ± 0.50 ^a^	<0.001
Docosahexaenoic acid	0.00	37.17 ± 4.33 ^b^	42.54 ± 3.70 ^b^	42.94 ± 3.03 ^b^	52.29 ± 4.09 ^a^	<0.001
Eicosapentaenoic acid	0.00	39.32 ± 3.71 ^b^	43.10 ± 3.40 ^b^	40.90 ± 4.60 ^b^	53.27 ± 4.24 ^a^	<0.001
Linoleic acid	355.43 ± 10.28 ^a^	75.10 ± 2.59 ^d^	284.66 ± 7.98 ^b^	191.92 ± 2.40 ^c^	176.52 ± 11.61 ^c^	<0.001
Linolenic acid	1434.66 ± 34.70 ^a^	73.89 ± 2.81 ^c^	133.57 ± 1.09 ^b^	128.62 ± 1.28 ^b^	135.64 ± 8.54 ^b^	<0.001

Note. The data were analyzed by ANOVA. Mean values ± standard deviations. Means in the same row with different superscripts are significant at the *p* < 0.05 level as determined by Tukey’s B test. Rows without superscript letters indicate no significant differences. 0.00 = not detected. Shaded values highlight diet-characteristic components: arachidic and heptadecanoic acid (MP only), EPA/DHA (all seabass-based diets), and lauric acid (SBB). “-” = not statistically compared (detected in only one diet). MP = mixed pollen diet, SB = seabass-based diet, SBM = seabass–mealworm-based diet, SBW = seabass–wax moth-based diet and SBB = seabass–black soldier fly-based diet.

## Data Availability

The original contributions presented in the study are included in the article; further inquiries can be directed to the corresponding author.

## Abbreviations

The following abbreviations are used in this manuscript:

AOAC	Association of Official Analytical Chemists
DHA	Docosahexaenoic acid
EAAs	Essential amino acids
EPA	Eicosapentaenoic acid
FAMEs	Fatty acid methyl esters
GC-FID	Gas Chromatography–Flame Ionization Detector
HCl	Hydrochloric acid
HPG	Hypopharyngeal gland
ICP-OES	Inductively Coupled Plasma–Optical Emission Spectrometry
LEfSe	Linear discriminant analysis effect size
MP	Mixed pollen-based diet
MUFAs	Monounsaturated fatty acids
NEAAs	Non-essential amino acids
PCA	Principal component analysis
PCR	Polymerase chain reaction
PUFAs	Polyunsaturated fatty acids
SB	Seabass-based diet
SBB	Seabass–black soldier fly-based diet
